# The Nonlinear Correlation Between a Novel Metabolic Score for Insulin Resistance and Subclinical Myocardial Injury in the General Population

**DOI:** 10.3389/fendo.2022.889379

**Published:** 2022-05-24

**Authors:** Zhenwei Wang, Wei Li, Jingjie Li, Naifeng Liu

**Affiliations:** ^1^ Department of Cardiology, Zhongda Hospital, School of Medicine, Southeast University, Nanjing, China; ^2^ Department of Cardiology, Affiliated Hospital of Yangzhou University, Yangzhou, China; ^3^ Department of Hematology and Oncology, Affiliated Xuchang People’s Hospital of Xinxiang Medical College, Xuchang, China

**Keywords:** insulin resistance, metabolic score for insulin resistance, subclinical myocardial injury, cardiovascular disease, NHANES III

## Abstract

**Background:**

Growing studies have shown that insulin resistance (IR) is associated with cardiovascular disease (CVD), while the association between IR and subclinical myocardial injury (SC-MI) remains unclear. Hence we aimed to assess the association between IR and SC-MI.

**Methods:**

In this cross-sectional study, we enrolled 6043 individuals (age: 58.43 ± 13.08 years; 46.2% men) free from CVD from the third National Health and Nutrition Examination Survey. A novel metabolic score for insulin resistance (METS-IR) was used as alternative markers of IR. Multivariate logistic regression and restricted cubic spline were performed to evaluate the associations between METS-IR and SC-MI.

**Results:**

The multivariate logistic regression analysis showed that after adjusting for cardiovascular metabolic risk factors, higher METS-IR was independently correlated with higher risk of SC-MI [as a quartile variable, Q4 vs Q1, OR (95% CI): 1.395 (1.147, 1.698), P = 0.001, P for trend < 0.001; as a continuous variable, per 10-unit increment, OR (95% CI): 1.869 (1.524, 2.292), P < 0.001]. Restricted cubic spline indicated that there was a J-curve connection between METS-IR and SC-MI. Threshold effect analysis ascertained an inflection point of 37 of METS-IR. The ORs (95% CIs) of per 10-unit increase of METS-IR for SC-MI were 0.707 (0.538, 0.928) and 1.327 (1.210, 1.456) on the left and right sides of the inflection point (P < 0.05), respectively. Subgroup analysis showed that the association between METS-IR and SC-MI was only statistically significant in participants without diabetes.

**Conclusions:**

METS-IR was nonlinearly related to SC-MI in the general population without CVD.

## Introduction

As an early asymptomatic myocardial injury, subclinical myocardial injury (SC-MI) is often hidden and easily overlooked in clinical work. It is reported that SC-MI can be diagnosed by a non-invasive, convenient and repeatable electrocardiograph (ECG) score, namely cardiac infarction/injury score (CIIS) (1). As a score designed to improve the accuracy of the diagnosis of myocardial injury, CIIS has been reported to have specificity of 95% and sensitivity of 85% in diagnosing myocardial infarction (1). Additionally, epidemiological studies have found that SC-MI was associated with the prevalence of cardiovascular disease (CVD) and increased all-cause and CVD-related deaths risks in the general population (2–4). Therefore, it can be seen that it is extremely important to control the occurrence and development of SC-MI.

It is reported that insulin resistance (IR) is involved in the occurrence of CVD (5). At present, IR can be evaluated by various methods. First of all, euglycaemic-hyperinsulinaemic clamp (EHC), as the gold standard for assessing IR, was first proposed in the 1970s (6). However, this method is difficult to be widely used because of its complex, expensive and invasive shortcomings. Secondly, triglyceride glucose index (TyG index), derived from fasting plasma glucose (FPG) and fasting triglycerides (TG), has currently become the most frequently used IR marker because of its low cost and easy availability (7, 8). Nevertheless, this index contains only two indicators of glucose and lipid metabolism, ignoring the role of cholesterol and nutritional status in CVD. Accordingly, TyG index may not fully reflect the cardiovascular effects of IR. Fortunately, Bello-Chavolla et al. recently developed a novel non-insulin-based metabolic score of IR, that is, METS-IR, combining FPG, TG, fasting high‐density lipoprotein cholesterol (HDL-C) and body mass index (BMI) mirroring nutritional status, which has been proved to be the powerful marker of IR outside EHC (9). METS-IR is reported to be related to many diseases, including diabetes (9), hypertension (10) and ischemic heart disease (11). Nonetheless, the association between METS-IR and SC-MI remains unknown.

Consequently, we aimed at investigating the association between METS-IR and SC-MI in this cross-sectional study.

## Materials and Methods

### Study Population

**Figure 1:**
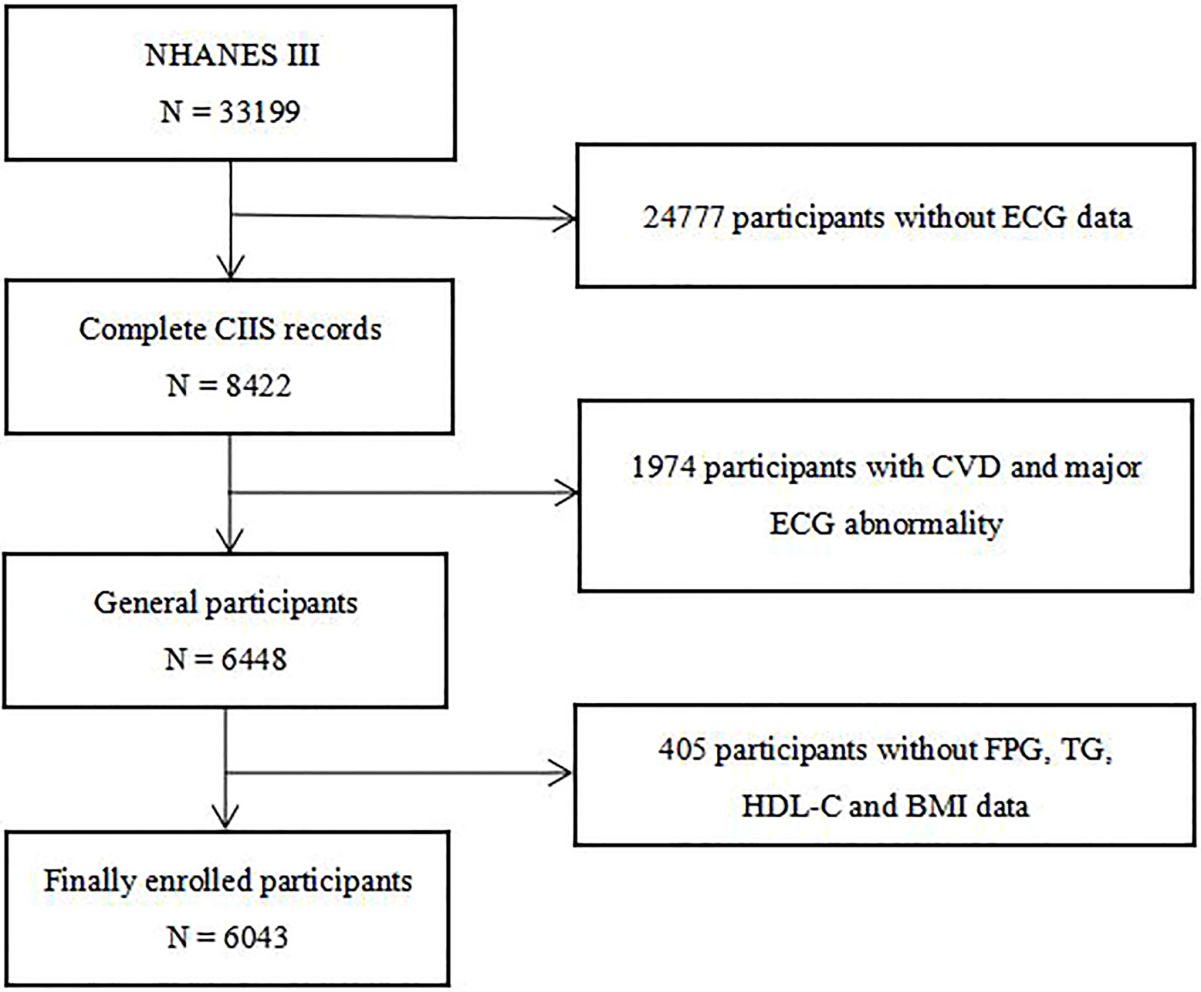
Flow chart of the study population enrollment. NHANES III the third National Health and Nutrition Examination Survey, ECG electrocardiograph, CIIS cardiac injury/infarction score, CVD cardiovascular disease, FPG fasting plasma glucose, TG triglycerides, HDL-C high-density lipoprotein cholesterol, BMI body mass index.

All participants in this study came from a national survey aimed at investigating the nutritional and health status of children and adults in the United States, namely the third National Health and Nutrition Examination Survey (NHANES III). After excluding individuals with CVD, major ECG abnormalities and absence of TG, FPG, HDL-C, BMI and CIIS data, 6043 individuals were finally enrolled in our study (**Figure 1**). Individuals in this study have provided written informed consent, and the study scheme was approved by the National Center for Health Statistics of the Center for Disease Control and Prevention Institutional Review Board and in line with the basic principles of the Declaration of Helsinki.

### Data Collection and Definitions

The staff of NHANES III collected demographic information from all participants through standardized questionnaires. Demographic variables enrolled in this study include age, sex, race, smoking history, prevalence of hypertension and diabetes. In this study, we divided races into four groups: non-Hispanic White, non-Hispanic Black, Mexican American and others. Those who claimed to have smoked more than 100 cigarettes were classified as smokers. The history of hypertension and diabetes were determined based on the self-reported situation of participants during the interview. The above-mentioned staff registered the BMI and blood pressure of each individual through standardized physical examination procedures. BMI was calculated according to the accepted formula, that is, weight (kg) divided by the square of height (m). Professionals of NHANES III measured the laboratory parameters of all individuals by standard biochemical analysis methods. The indicators used for this study included FPG, hemoglobin A1c (HbA1c), TG, TC, HDL-C, low-density lipoprotein cholesterol (LDL-C), uric acid (UA), blood urea nitrogen (BUN), fibrinogen, creatinine and C-reactive protein (CRP).

METS-IR was calculated on the basis of the previously published formula, that is, METS-IR = ln(2 × FPG [mg/dL] + TG [mg/dL]) × BMI [kg/m^2^]/ln(HDL-C [mg/dL]), in which the blood indicators were derived from the venous blood of participants who fasted for more than 8 hours (9). The TyG index was calculated as ln[TG (mg/dl)*FPG (mg/dl)/2] (7). The TG/HDL-C was calculated as TG-to-HDL-C ratio.

SC-MI was obtained by professionals through the multivariable decision theory electrocardiogram (ECG) classification scheme, the specific details of which were available elsewhere (1). In short, professionals constructed a risk hierarchical scoring system based on objective ECG waveform components that might be related to myocardial injury or ischemia, and then counted the combination of 4 continuous and 11 discrete features, and finally determined the total score for evaluating the severity of myocardial injury (1). According to previous studies, we define SC-MI as CIIS ≥ 10 (1, 4).

### Statistical Analysis

Continuous variables were showed as mean ± standard deviation or median (first quartile, third quartile), and categorical variables were reported as frequencies (percentages). Using one-way ANOVA or Kruskal-Wallis H test for continuous variables and Chi-square or Fisher’s exact test for categorical variables to compare the differences between groups. Performing univariate and multivariate logistic regression models to evaluate the associations between METS-IR, TyG, TG/HDL-C and SC-MI. Model 1: adjusted for age and sex; Model 2: adjusted for variables included in Model 1 and race, smoking, diabetes, hypertension; Model 3: adjusted for variables included in Model 2 and systolic blood pressure (SBP), diastolic blood pressure (DBP), TC, fibrinogen, CRP, creatinine, UA, BUN, HbA1c; Model 4: adjusted for variables included in Model 3 and BMI, TG, HDL-C, FPG. Additionally, we used the restricted cubic splines with 3 knots at 10th, 50th, and 90th percentage with adjustment for variables included in Model 4 to fit the nonlinear relationship between METS-IR and SC-MI, and determined the inflection point of threshold effect of METS-IR for SC-MI, and compared the 1-line logistic regression model with 2-piecewise logistic regression model by log-likelihood ratio test. And we performed a 2-piecewise multivariable linear regression analysis with variables analyzed in logistic analysis to estimate the independent contribution of METS-IR on CIIS. Additionally, we conducted a subgroup analysis in multivariable logistic regression analysis to explore the stratified association between METS-IR and SC-MI based on different subgroup of diabetes, and the covariates of the full-adjusted model in the subgroup analysis did not include the stratified variable. All Statistical analyses were performed by using SPSS 19.0 (SPSS Inc., Chicago, Illinois, USA) and R Programming Language (version 3.6.3). A two-tailed P value < 0.05 was regarded as statistically significant.

## Results

### Baseline Characteristics of Study Participants

6043 participants (mean age: 58.43 ± 13.08 years; 46.2% men) were divided into 4 groups by the quartile of the METS-IR: Q1: < 34.36, Q2: 34.36-40.69, Q3: 40.69-47.93, Q4: ≥ 47.93. Compared with individuals with lower METS-IR, individuals with higher METS-IR had higher age and higher prevalence of hypertension, diabetes and SC-MI, and were more likely to be men and Mexican-American (P < 0.001). In terms of cardiovascular metabolic risk factors, individuals with higher METS-IR had higher levels of BMI, SBP, TG, TC, LDL-C, fibrinogen, CRP, UA, creatinine, FPG, HbA1c and lower level of HDL-C (P < 0.001) (**Table 1**).

**Table 1:** Baseline characteristics of participants stratified by the quartile of the METS‐IR.

	Total population	Q1	Q2	Q3	Q4	P value
N	6043	1510	1509	1513	1511	
Age, years	58.43 ± 13.08	59.45 ± 14.12	59.17 ± 13.30	58.68 ± 13.02	56.42 ± 11.55	<0.001
Sex, male, n (%)	2790 (46.2)	542 (35.9)	753 (49.9)	756 (50.0)	739 (48.9)	<0.001
Race, n (%)						<0.001
Non-Hispanic white	3074 (50.9)	886 (58.7)	804 (53.3)	728 (48.2)	656 (43.4)	
Non-Hispanic black	1296 (21.4)	320 (21.2)	319 (21.1)	331 (21.9)	326 (21.6)	
Mexican-American	1416 (23.4)	234 (15.5)	319 (21.1)	386 (25.5)	477 (31.6)	
Others	257 (4.3)	70 (4.6)	67 (4.4)	68 (4.5)	52 (3.4)	
Smoking, n (%)	3280 (54.3)	833 (55.2)	832 (55.1)	823 (54.4)	792 (52.4)	0.384
Diabetes, n (%)	599 (9.9)	60 (4.0)	99 (6.6)	153 (10.1)	287 (19.0)	<0.001
Hypertension, n (%)	1939 (32.2)	305 (20.3)	434 (28.9)	532 (35.3)	668 (44.3)	<0.001
Body mass index, kg/m^2^	27.61± 5.46	22.08 ± 2.19	25.73 ± 2.04	28.60 ± 2.49	34.01 ± 5.29	<0.001
SBP, mmHg	131.05 ± 18.96	127.74 ± 20.19	130.16 ± 19.01	132.71 ± 18.63	133.60 ± 17.35	<0.001
Triglycerides, mg/dL	127.0 (90.0, 183.0)	88 (69.0, 115.0)	118.0 (89.0, 156.0)	146.0 (107.0, 198.0)	185.5 (131.0, 269.0)	<0.001
Total cholesterol, mg/dL	217.54 ± 42.94	211.96 ± 41.11	218.89 ± 42.09	220.05 ± 42.66	219.26 ± 45.34	<0.001
LDL−C, mg/dL	136.35 ± 38.30	125.86 ± 38.43	140.12 ± 37.11	142.05 ± 37.25	137.64 ± 38.34	<0.001
HDL−C, mg/dL	51.52 ± 16.46	65.36 ± 18.03	53.13 ± 13.05	46.94 ± 11.64	40.68 ± 10.99	<0.001
Fibrinogen, mg/dL	309.36 ± 84.91	297.86 ± 79.88	305.45 ± 86.98	312.81 ± 83.41	321.13 ± 87.41	<0.001
C-reactive protein, mg/dL	0.21 (0.21, 0.50)	0.21 (0.21, 0.21)	0.21 (0.21, 0.33)	0.21 (0.21, 0.53)	0.33 (0.21, 0.77)	<0.001
Creatinine, mg/dL	1.09 ± 0.32	1.05 ± 0.27	1.11 ± 0.39	1.10 ± 0.37	1.11 ± 0.37	<0.001
Uric acid, mg/dL	5.39 ± 1.45	4.75 ± 1.32	5.26 ± 1.36	5.60 ± 1.37	5.95 ± 1.48	<0.001
BUN, mg/dL	15.17 ± 5.55	14.86 ± 5.85	15.23 ± 5.40	15.22 ± 5.22	15.37 ± 5.68	0.073
FPG, mg/dL	95.0 (88.0, 105.0)	90.0 (85.0, 97.0)	94.0 (87.0, 102.0)	97.0 (90.0, 107.0)	101.0 (92.0, 120.0)	<0.001
HbA1c, %	5.74 ± 1.22	5.36 ± 0.63	5.55 ± 0.90	5.80 ± 1.28	6.25 ± 1.64	<0.001
METS-IR	42.00 ± 10.69	30.03 ± 3.07	37.58 ± 1.80	44.07 ± 2.04	56.32 ± 8.28	<0.001
CIIS	2.2 (0, 8.8)	2.5 (0, 8.7)	2.0 (0, 8.0)	1.5 (0, 7.9)	3.1 (0, 10.3)	<0.001
SC-MI, n (%)	1302 (21.5)	311 (20.6)	311 (20.6)	284 (18.8)	396 (26.3)	<0.001

Data are expressed as mean ± SD, median (first quartile, third quartile), or n (%). METS-IR, metabolic score for insulin resistance; SBP, systolic blood pressure; LDL-C, low-density lipoprotein cholesterol; HDL-C, high-density lipoprotein cholesterol; BUN, blood urea nitrogen; FPG, fasting plasma glucose; HbA1c hemoglobin A1c; CIIS, cardiac infarction/injury score; SC-MI, subclinical myocardial injury.

### Associations Between METS-IR, TyG, TG/HDL-C and SC-MI

The multivariable logistic regression analyses results of the associations between METS-IR, TyG, TG/HDL-C and SC-MI were displayed in **Table 2**. After fully adjusting for confounding covariates including age, sex, race, smoking, diabetes, hypertension, SBP, DBP, TC, fibrinogen, CRP, creatinine, UA, BUN, HbA1c, BMI, TG, HDL-C and FPG, whether as a continuous variable or classified variable, the higher METS-IR was independently related to the higher risk of SC-MI [as a classified variable, Q4 vs Q1, OR (95% CI): 1.395 (1.147, 1.698), P = 0.001, P for trend < 0.001; as a continuous variable, per 10-unit increment, OR (95% CI): 1.869 (1.524, 2.292), P < 0.001]. Additionally, we also found that with the increase of confounding factors, the association between TyG and SC-MI was no longer statistically significant (P for trend > 0.05), while the association between TG/HDL-C and SC-MI was weakened [Q4 vs Q1, OR (95% CI): 1.310 (1.075, 1.597), P = 0.007].

**Table 2:** Multivariate logistic regression analyses of associations between METS-IR, TyG, TG/HDL-C and SC-MI.

	Model 1		Model 2		Model 3		Model 4	
	OR (95% CI)	P value	OR (95% CI)	P value	OR (95% CI)	P value	OR (95% CI)	P value
METS-IR								
Q1	Ref	-	Ref	-	Ref	-	Ref	-
Q2	0.982 (0.820, 1.175)	0.843	0.982 (0.819, 1.178)	0.848	1.008 (0.833, 1.220)	0.934	1.008 (0.833, 1.220)	0.934
Q3	0.885 (0.737, 1.063)	0.191	0.872 (0.723, 1.050)	0.149	0.883 (0.726, 1.075)	0.216	0.883 (0.726, 1.075)	0.216
Q4	1.482 (1.246, 1.763)	<0.001	1.438 (1.197, 1.727)	<0.001	1.395 (1.147, 1.698)	0.001	1.395 (1.147, 1.698)	0.001
P for trend	-	<0.001	-	<0.001	-	<0.001	-	<0.001
METS‐IR^a^	1.016 (1.011, 1.022)	<0.001	1.016 (1.010, 1.022)	<0.001	1.013 (1.006, 1.020)	<0.001	1.065 (1.043, 1.086)	<0.001
METS‐IR^b^	1.177 (1.111, 1.247)	<0.001	1.173 (1.105, 1.245)	<0.001	1.137 (1.065, 1.215)	<0.001	1.869 (1.524, 2.292)	<0.001
TyG								
per 1-unit increase	1.262 (1.149, 1.386)	<0.001	1.267 (1.149, 1.396)	<0.001	1.145 (1.014, 1.292)	0.028	1.145 (1.014, 1.292)	0.028
Q1	Ref	-	Ref	-	Ref	-	Ref	-
Q2	0.859 (0.714, 1.034)	0.108	0.871 (0.722, 1.050)	0.148	0.857 (0.703, 1.045)	0.127	0.835 (0.681, 1.025)	0.084
Q3	1.050 (0.878, 1.255)	0.593	1.050 (0.875, 1.261)	0.598	1.003 (0.823, 1.222)	0.976	0.956 (0.770, 1.187)	0.682
Q4	1.296 (1.089, 1.542)	0.004	1.314 (1.097, 1.574)	0.003	1.112 (0.895, 1.381)	0.340	1.024 (0.773, 1.357)	0.869
P for trend	-	<0.001	-	<0.001	-	0.094	-	0.212
TG/HDL-C								
per 1-unit increase	1.032 (1.017, 1.047)	<0.001	1.030 (1.014, 1.046)	<0.001	1.023 (1.007, 1.040)	0.005	1.023 (1.007, 1.040)	0.005
Q1	Ref	-	Ref	-	Ref	-	Ref	-
Q2	0.912 (0.760, 1.095)	0.323	0.931 (0.774, 1.119)	0.444	0.895 (0.737, 1.087)	0.262	0.895 (0.737, 1.087)	0.262
Q3	0.948 (0.791, 1.136)	0.560	0.960 (0.798, 1.154)	0.665	0.935 (0.769, 1.137)	0.501	0.935 (0.769, 1.137)	0.501
Q4	1.389 (1.166, 1.653)	<0.001	1.428 (1.194, 1.709)	<0.001	1.310 (1.075, 1.597)	0.007	1.310 (1.075, 1.597)	0.007
P for trend	-	<0.001	-	<0.001	-	<0.001	-	<0.001

Model 1: adjusted for age and sex; Model 2: adjusted for variables included in Model 1 and race, smoking, diabetes, hypertension; Model 3: adjusted for variables included in Model 2 and systolic blood pressure, diastolic blood pressure, total cholesterol, fibrinogen, c-reactive protein, creatinine, uric acid, blood urea nitrogen, hemoglobin A1c.

Model 4: adjusted for variables included in Model 3 and body mass index, triglycerides, high-density lipoprotein cholesterol, fasting plasma glucose.

METS-IR, metabolic score for insulin resistance; SC-MI, subclinical myocardial injury; TyG, triglyceride glucose index; TG/HDL-C, triglycerides/high-density lipoprotein cholesterol; OR, odd ratio; CI, confidence interval.

a The OR was examined by per 1-unit increase of METS‐IR.

b The OR was examined by per 10-unit increase of METS‐IR.

**Figure 2:**
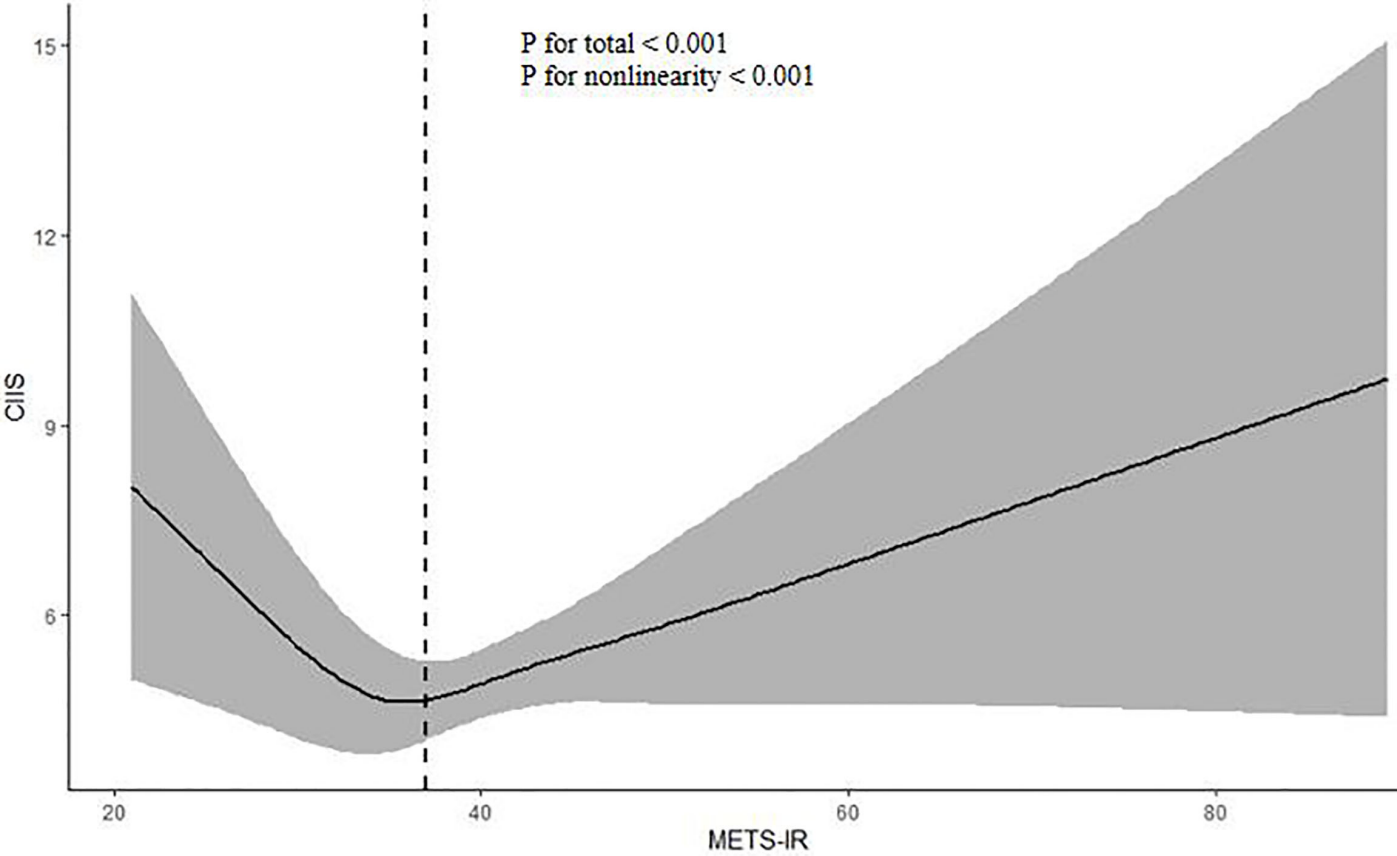
Restricted cubic spline plot of the association between METS-IR and CIIS. The association was adjusted for variables included in Model 4. *METS-IR* metabolic score for insulin resistance, *CIIS* cardiac infarction/injury score.

The results of restricted cubic spline analysis showed that there was a significant nonlinear J-curve correlation between METS-IR and SC-MI (P for nonlinearity < 0.001) (**Figure 2**). The subsequent threshold effect analysis determined that the inflection point of METS-IR was 37.0. Segmented logistic regression analysis exhibited that when METS-IR ≤ 37.0, for each 10 units increment of METS-IR, the prevalence of SC-MI decreased by 29.3%, whereas when METS-IR > 37.0, the prevalence of SC-MI increased by 32.7% (OR 0.707, 95% CI 0.538-0.928, P < 0.05; OR 1.327, 95% CI 1.210-1.456, P < 0.001; respectively) (**Table 3**). And we found that the segmented logistic regression model was better than the l-line logistic regression model for fitting the association between METS-IR and SC-MI.

**Table 3:** Threshold effect of METS-IR on SC-MI using piecewise binary logistic regression models.

	Inflection point	Group	OR (95% CI)	P for log likelihood ratio test
METS-IR^a^	37	≤ 37	0.966 (0.940, 0.993)*	<0.05
		> 37	1.029 (1.019, 1.038)**	
METS-IR^b^	3.7	≤ 3.7	0.707 (0.538, 0.928)*	<0.05
		> 3.7	1.327 (1.210, 1.456)**	

a The OR was examined by per 1-unit increase of METS‐IR.

b The OR was examined by per 10-unit increase of METS‐IR. Analyses was adjusted for variables included in Model 4. *P < 0.05, **P < 0.001. METS-IR, metabolic score for insulin resistance; SC-MI, subclinical myocardial injury; OR, odd ratio; CI, confidence interval.

### Association Between METS-IR and CIIS

The association between METS-IR and CIIS was nonlinear in the total population, while restricted cubic spline plot showed that when METS-IR ≤ 37, METS-IR was negatively associated with CIIS, while when METS-IR > 37, this association was positive, so we carried out piecewise multivariate linear regression analyses to explore the independent contribution of METS-IR on CIIS. The results showed that after gradually adjusting for confounding factors, when METS-IR ≤ 37, the CIIS decreased by 1.31-1.48 for each unit increase of METS-IR, while when METS-IR > 37, the CIIS increased by 0.71-0.91 for each unit increase of METS-IR (P < 0.001) (**Table 4**).

**Table 4:** Multivariable linear regression between METS-IR and CIIS.

	METS-IR ≤ 37		METS-IR > 37	
	*β* (95% CI)	P value	*β* (95% CI)	P value
Model 1	-1.446 (-2.128, -0.763)	<0.001	0.908 (0.656, 1.159)	<0.001
Model 2	-1.308 (-1.994, -0.623)	<0.001	0.798 (0.540, 1.057)	<0.001
Model 3	-1.477 (-2.209, -0.746)	<0.001	0.705 (0.416, 0.994)	<0.001

Model 1: adjusted for age and sex; Model 2: adjusted for variables included in Model 1 and race, smoking, diabetes, hypertension; Model 3: adjusted for variables included in Model 2 and systolic blood pressure, diastolic blood pressure, total cholesterol, fibrinogen, c-reactive protein, creatinine, uric acid, blood urea nitrogen, hemoglobin A1c. METS-IR, metabolic score for insulin resistance; CIIS, cardiac infarction/injury score; CI, confidence interval.

### Subgroup Analysis Between METS-IR and SC-MI

We performed a subgroup analysis to assess the potential factors modifying the association between METS-IR and SC-MI and found that the association between METS-IR and SC-MI was only statistically significant in participants without diabetes [Q4 vs Q1, OR (95% CI): 1.436 (1.157, 1.784), P = 0.001, P for trend < 0.001, P for interaction > 0.05] (**Table 5**).

**Table 5:** Subgroups analysis for the association between METS-IR and SC-MI.

	Q1	Q2	Q3	Q4		
	OR (95% CI)	OR (95% CI)	OR (95% CI)	OR (95% CI)	P-t	P-int
Diabetes						0.893
Yes	Ref	0.468 (0.210, 1.043)	0.443 (0.208, 0.944)*	0.643 (0.305, 1.356)	0.112	
No	Ref	1.051 (0.863, 1.281)	0.898 (0.728, 1.106)	1.436 (1.157, 1.784)**	<0.001	

The model was adjusted for age, sex, race, smoking, hypertension, systolic blood pressure, diastolic blood pressure, total cholesterol, fibrinogen, c-reactive protein, creatinine, uric acid, blood urea nitrogen, hemoglobin A1c. METS-IR, metabolic score for insulin resistance; SC-MI, subclinical myocardial injury; P-t, P for trend; P-int, P for interaction; OR, odd ratio; CI, confidence. *P < 0.05, **P < 0.01.

## Discussion

Our study was the first report on the association between METS-IR and SC-MI. The findings showed that there was an independent J-type correlation between METS-IR and SC-MI. On the left side of inflection point, the risk of SC-MI decreased with the increase of METS-IR, while on the right side of the inflection point, the risk of SC-MI increased with the increase of METS-IR.

It is well known that IR, as the core component of metabolic syndrome, is also a risk factor for metabolism-related diseases. At present, a large number of studies have focused on the effects of IR on metabolism-related diseases. Nevertheless, there are many kinds of markers of IR, so it is difficult to determine which marker has the best performance in predicting these diseases. EHC is considered to be the gold standard for the diagnosis of IR, while it has been gradually abandoned because of its complex and expensive shortcomings (6). Secondly, the homeostasis model assessment for IR (HOMA-IR), which is viewed as the silver standard, is limited in epidemiological studies because of its dependence on insulin indicator (12). Conversely, TyG index and TG/HDL-C, which only contain blood routine indicators, were once widely used in epidemiological studies, and were considered as cheap, convenient, easily available and highly applicable alternative indicators of IR (13–17). However, evidences suggest that obesity or higher BMI, which reflects overnutrition, is also a risk factor of IR (18, 19), so the above index may not fully represent IR because of the lack of nutritional indicators. Given the above situation, a novel non-insulin-based metabolic score for IR has recently been developed, that is, METS-IR derived from clinical routine parameters including FPG, TG, HDL-C and BMI, which includes not only glucose and lipid metabolism indicators but also nutritional index, and has been widely used in epidemiological studies (9, 10, 20). For example, Bello-Chavolla et al. compared the superiority of METS-IR and other IR indexes including EHC, TyG and TG/HDL-C in diagnosing impaired insulin sensitivity in a sample containing EHC data, the results showed that METS-IR was superior to EHC and other markers of IR, a subsequent prospective validation cohort study involving 6,144 participants displayed that participants in the highest quartile of METS-IR had an approximately 4 times higher risk of developing diabetes than those in the lowest quartile (HR: 3.91,95% CI:2.25-6.81), which was the first report to prove that METS-IR could be used to screen insulin sensitivity and metabolic related diseases (9). Subsequently, Liu et al. found an independent positive correlation between METS-IR and blood pressure levels in a large epidemiological cohort of 142,005 adults who did not take antihypertensive drugs, and that higher METS-IR was independently associated with higher risk of hypertension, which was also robust in gender subgroups (10). Besides, some studies have shown that METS-IR was associated with uric acid level, prehypertension, arterial stiffness, early renal dysfunction, ischemic heart disease and metabolic syndrome (11, 20–27). Likewise, our study also obtained similar results, that is, METS-IR was independently related to SC-MI, while this correlation was nonlinear. When METS-IR ≤ 37.0, the risk of SC-MI decreased with the increase of METS-IR, whereas the risk of SC-MI increased with the increase of METS-IR when METS-IR > 37.0. The reason for this might be that both malnutrition and overnutrition contributed to myocardial damage (28). To sum up, METS-IR may be an economical and convenient screening index for CVD.

Though we proved the association between METS-IR and SC-MI, the mechanism remained unknown. Based on the published literature, we found that there might be several potential mechanisms that mediated their association. For instance, there was evidence that IR promoted visceral obesity, dyslipidemia, endothelial dysfunction and elevated inflammatory markers, which were also risk factors for myocardial injury (29). Furthermore, Ding et al. also found that METS-IR was positively correlated with inflammatory activity and disorder of adipose factors, which might be involved in the occurrence of myocardial injury (30). Finally, due to the involvement of BMI, METS-IR might be a better indicator of IR in adipose tissue, muscle and liver, and might also play a more important role during the occurrence of myocardial injury (31). However, the above mechanisms were only used to explain the harmful risks of IR, while we accidentally found that IR had a potential protective effect on the risk of SC-MI in a certain range. Nevertheless, this unexpected result was not necessarily wrong. Previous studies have found that impaired insulin signal could prolong the life expectancy of worms, flies, mice and Caenorhabditis elegans, and showed that specific insulin receptor gene mutations could resist aging and oxidative stress (32–34). However, it was unknown whether this findings could apply to humans. In addition, there was evidence that IR caused by mutations in the insulin/insulin-like growth factor-1 signaling pathway failed to affect their life expectancy in different ethnic groups (35). Perhaps in some cases, IR can be used as a potential protective mechanism to combat metabolic disorders and enhance the defense ability of cells. However, there are many factors that can cause or promote the occurrence of IR, among which obesity plays a very important role in the occurrence and development of IR. In this study, we explored the association between METS-IR, an alternative marker of IR including BMI, and SC-MI, and unexpectedly found that there was a nonlinear association between METS-IR and SC-MI. METS-IR below the threshold could reduce the risk of SC-MI. In other words, individuals below the threshold represented ordinary people with normal nutrition, and the closer they got to the threshold, the better the their nutritional status and glucose and lipid metabolism, while the harmful effects of IR in these people were weakened, so they have a lower risk of developing SC-MI. However, more studies were warranted to explore the underlying mechanisms.

Although this study acquired unexpected findings, there were still several uncontrollable limitations. For example, we failed to identify the causal association between independent and dependent variable in this cross-sectional study. In addition, there were presently a variety of markers of IR, including insulin-derived and non-insulin-derived indices, while our study mainly explored the association between METS-IR and SC-MI and failed to compare which marker was more superior in the diagnosis of SC-MI in this population. Additionally, there might be other confounding factors, such as diet and drugs. Finally, only adults from the United States were enrolled in this study, consequently, the findings might not apply to other countries and populations.

In summary, this study confirmed that METS-IR, a novel non-insulin-based metabolic score for IR, was nonlinearly related to SC-MI, which further highlighted the role of IR in the occurrence and development of CVD.

## Data Availability Statement

Publicly available datasets were analyzed in this study. This data can be found here: https://wwwn.cdc.gov/nchs/nhanes/Default.aspx.

## Ethics Statement

The studies involving human participants were reviewed and approved by National Center for Health Statistics of the Center for Disease Control and Prevention Institutional Review Board. The patients/participants provided their written informed consent to participate in this study.

## Author Contributions

ZW and WL conceived and designed the study. WL and JL were responsible for the management and retrieval of data, contributed to initial data analysis and interpretation. ZW drafted the initial manuscript. NL revised the manuscript. NL was the guarantor of this work and had full access to all the data in the study and take responsibility for its integrity and the accuracy of the data analysis. All authors read and approved the final manuscript.

## Conflict of Interest

The authors declare that the research was conducted in the absence of any commercial or financial relationships that could be construed as a potential conflict of interest.

## Publisher’s Note

All claims expressed in this article are solely those of the authors and do not necessarily represent those of their affiliated organizations, or those of the publisher, the editors and the reviewers. Any product that may be evaluated in this article, or claim that may be made by its manufacturer, is not guaranteed or endorsed by the publisher.
